# DeepFoG: An IMU-Based Detection of Freezing of Gait Episodes in Parkinson’s Disease Patients via Deep Learning

**DOI:** 10.3389/frobt.2021.537384

**Published:** 2021-05-07

**Authors:** Thomas Bikias, Dimitrios Iakovakis, Stelios Hadjidimitriou, Vasileios Charisis, Leontios J. Hadjileontiadis

**Affiliations:** ^1^ Department of Electrical and Computer Engineering, Aristotle University of Thessaloniki, Thessaloniki, Greece; ^2^ Department of Electrical and Computer Engineering, Khalifa University of Science and Technology, Abu Dhabi, United Arab Emirates

**Keywords:** Parkinson's disease, freezing of gait, deepFoG, smartwatch, deep learning, rhythmic auditory stimulation

## Abstract

Freezing of Gait (FoG) is a movement disorder that mostly appears in the late stages of Parkinson’s Disease (PD). It causes incapability of walking, despite the PD patient’s intention, resulting in loss of coordination that increases the risk of falls and injuries and severely affects the PD patient’s quality of life. Stress, emotional stimulus, and multitasking have been encountered to be associated with the appearance of FoG episodes, while the patient’s functionality and self-confidence are constantly deteriorating. This study suggests a non-invasive method for detecting FoG episodes, by analyzing inertial measurement unit (IMU) data. Specifically, accelerometer and gyroscope data from 11 PD subjects, as captured from a single wrist-worn IMU sensor during continuous walking, are processed via Deep Learning for window-based detection of the FoG events. The proposed approach, namely DeepFoG, was evaluated in a Leave-One-Subject-Out (LOSO) cross-validation (CV) and 10-fold CV fashion schemes against its ability to correctly estimate the existence or not of a FoG episode at each data window. Experimental results have shown that DeepFoG performs satisfactorily, as it achieves 83%/88% and 86%/90% sensitivity/specificity, for LOSO CV and 10-fold CV schemes, respectively. The promising performance of the proposed DeepFoG reveals the potentiality of single-arm IMU-based real-time FoG detection that could guide effective interventions via stimuli, such as rhythmic auditory stimulation (RAS) and hand vibration. In this way, DeepFoG may scaffold the elimination of risk of falls in PD patients, sustaining their quality of life in everyday living activities.

## 1 Introduction

Parkinson’s Disease (PD) is a progressive neurological disorder related to multiple motor symptoms which affect patients’ movement and stability. One of the most common and most disturbing symptoms is Freezing of Gait (FoG) (Giladi et al., 2001). FoG is characterized by the inability to walk through narrow corridors or to take short and fast steps. This results in difficulty in initiating gait or turning while walking, despite the intention of the patient (Nutt et al., 2011; Heremans et al., 2013). Although FoG episodes usually make their presence in the late stages of PD or other Parkinsonian syndromes, in cases where the motor symptoms are already intense, FoG episodes may also occur in early stages (Hall et al., 2015). The latter is due to the lack of timely medication. The pathological hallmark of FoG points to disturbances in the frontal cortical regions, the basal ganglia, and the midbrain locomotor region which are the most probable origins (Nutt et al., 2011). FoG has been associated with cerebellum functionality (Fasano et al., 2017) and has been often described as a derangement of the cognitive dynamics (Pozzi et al., 2019). Moreover, occurrence of FoG has been interdependent on the emotional condition of the PD patient, anxiety and/or stress levels (Witt et al., 2019). Despite the increasing research interest in finding the exact causes and characteristics of FoG events, the biological and neurological nature of FoG has not been fully decoded. Importantly, deep understanding of FoG is significantly impeded by the fact that the profile of FoG may differ substantially amongst patients.

Manifestation of FoG episodes harshly downgrades PD patients’ Quality-of-Life (QoL) and affects their mental condition and self-esteem. Concretely, PD patients are restricted from performing essential functions, such as walking initiation and direction change. Furthermore, they experience mood alterations which gradually lead to serious mental health issues. Together with emotional swings, the greater risk of living with FoG is the sudden and instantaneous akinesia, which raises the possibility of falls and injuries (Bloem et al., 2004). This problem is intensified in the case of the elderly PD patients, and especially those who are unattended, wherein their bones are more vulnerable, and hence the chances of FoG occurrences are much higher.

The confrontation of FoG events is an essential necessity that concerns the medical society. Multiple solutions have been proposed to eliminate the FoG symptoms. Medication treatment, including use of levodopa (Fietzek et al., 2013) or monoamine oxidase B inhibitor, rasagiline (Coria and Cozar-Santiago, 2008), is commonly provided to deal with the severity of the motor symptoms and, in particular, FoG episodes. Although several potential drug treatments have been suggested (Zhang et al., 2016), pharmaceutical intake frequently fails to alleviate the symptoms, while, in some cases, its effect lasts only during the drug-ON period. Moreover, medication may very often cause side effects (Hauser, 2009) which lead to further inconveniences to patients. Finally, the estimated expenses of medication are rather high and can become a significant financial burden. Invasive solution of Deep brain stimulation has also been introduced in the event of medication failure or in the extreme condition of total inability to walk (Huang et al., 2018). Nevertheless, the neurological surgery is rarely recommended due to the high risk of dangerous or fatal side effects caused by the direct cerebral intervention.

Alternative studies propose improvement of FoG episodes’ characteristics through cognitive exercises (Walton et al., 2018). However, given that the PD patients vary in their cultural, educational, and cognitive characteristics, tailored and personalized cognitive exercise should be considered. Exercises and training with rhythmic cue have indicated that FoG episodes can be countered when patients synchronize their movement with external stimulus (Allen et al., 2010). Rhythmic Auditory Stimulation (RAS) constitutes a solution that may be acceptable for the majority of PD patients. RAS offers improvement in gait (Dalla Bella et al., 2017) via synchronization of the patient’s steps with an external acoustic cuing that helps to overcome the failure of walking initiation. In addition, it has been observed that PD patients are less likely to encounter FoG episodes when they walk in coordination with stimulus (Thaut et al., 1996; Lim et al., 2005; Hausdorff et al., 2007). Currently, such solutions require the physical presence of physicians and medical staff. However, application of RAS outside medical sites would allow for restoring patients’ independence, while would limit the required Healthcare resources. This would be a significant step forward towards the effective facilitation of the increasing number of PD patients (Dorsey et al., 2007). At large, detection of the FoG events is crucial to be performed timely, and, therefore, a real-time RAS process could be valuable for eliminating FoG *per se* and its related risks.

In this view, multiple research groups have investigated Fog detection using methods which rely on sensor placement in different parts of the patient’s body. Essentially, these methods can be divided into two categories based on whether a sensor is placed on the upper limb of the patient or not. Jovanov et al. (2009); Niazmand et al. (2011); Bachlin et al. (2009) have suggested the use of multiple devices attached on the lower and middle body to capture FoG events, using accelerometer and gyroscope sensors. Mazilu et al. (2015a) proposed a gait-assist system with sensors worn on both ankles to detect FoG events from four Fast Fourier Transformation (FFT) features by using Machine Learning. Overall, the majority of prior work utilizes measuring devices which are allocated on the ankles, the hips or the waist of the patient (Table 1).

**TABLE 1:** Comparison of the proposed single-wrist approach FoG detection with other studies exploiting one or more sensors to capture movement.

	Sensors	Sensors placement	Realtime	Results
*Without sensor in upper limb*				
Bachlin et al. (2009)	3	Ankle, thigh and lower back	Yes	73.1% sensitivity
				81.6% specificity
Jovanov et al. (2009)	2	Both sensors on belt or knee or ankle or shoe	Yes	unknown
Niazmand et al. (2011)	5	Shank and belt	No	88.3% sensitivity
				85.3% specificity
Mazilu et al. (2015a)	2	Ankles	Yes	97% hit rate
Palmerini et al. (2017)	3	Shins, lower back	Yes	83% sensitivity
				67% specificity
Camps et al. (2018)	1	Waist	Yes	92.6% sensitivity
				88.7% specificity
*With sensor in upper limb*				
Cole et al. (2011)	3	Shank, thigh and arm	No	83% sensitivity
				97% specificity
Tripoliti et al. (2013)	6	Wrists, ankles, waist and chest	No	81.9% sensitivity
				98.7% specificity
Mazilu et al. (2015b)	2	Both wrists	Yes	90% sensitivity
				83% specificity
DeepFoG	1	Wrist	Yes	83% sensitivity
				88% specificity

Contrary to the aforementioned works, other studies (Ziv et al. (1999); Vercruysse et al. (2012)) have reported a significant correlation between FoG episodes and upper limb motions. In this respect, various methods have been introduced for detecting FoG episodes from sensors placed on the upper limbs. Specifically, Cole et al. (2011) exploited accelerometer sensors attached on the arm, the shank, and the thigh, along with Electromyography sensors (EMG) to compute to detect instances of FoG episodes using Dynamic Neural Networks. Their method achieved 83% sensitivity and 97% specificity. In another study, Tripoliti et al. (2013) have associated FoG events with wrist movement. Despite of the satisfactory accuracy (82%/90% sensitivity/specificity), their method required the use of five additional sensors (worn in ankles, waist, and chest), leading to inconvenience and thus low user acceptance.

Installation of devices in multiple body parts is rather doubtful due to the inconvenience caused by the volume, weight, difficulty in placement of the hardware and/or discomfort in movement. Exclusive association between FoG and wrist movement has been proposed by Mazilu et al. (2015b) who introduced a Machine Learning model to distinguish FoG episodes from other events during walking (i.e., turns, stops, obstacle avoidance). They used time and frequency features from accelerometer and gyroscope data using sensors attached at both wrists. Their classification pipeline achieved 90%/83% sensitivity/specificity.

Consequently, exploiting the increasing rise of smartwatch sensors, the present study investigates the feasibility of a single wrist-based inertial measurement unit (IMU) FoG classification, namely, DeepFoG, for facilitating the real-time detection of FoG episodes. The initial hypothesis is that data collected only from one wrist should provide sufficient information for effectively predicting FoG events. We proposed a non-invasive user-centric solution that can be easily applied in everyday life. Given that FoG is a complex activity task and the research knowledge on this domain is still being explored, manual feature engineering would be rather complicated, and thus application of Deep Learning could be suitable for single-wrist detection (Wang et al., 2019). DeepFoG is based on the training of a Deep Learning model that automatically detects FoG events and differentiates them from stops and walking with turns. The ecological validity of DeepFoG aims towards achieving higher user acceptance, while providing comparative detection performance when compared to the prior art.

## 2 Materials and Methods

A Deep Learning approach is being proposed for the detection of FoG events from stops and turns during gait. Our proposed framework is based on linear and angular acceleration data from one wrist that are used as input to a Convolutional Neural Network (CNN). The CNN is trained and evaluated using the CuPiD dataset (Mazilu et al., 2012). These data are fed to the CNN classifier to detect FoG episodes (Class 1) from Walking-with-Turns (Class 2) and Stops (Class 3) in a sliding window manner. Firstly, sensitivity and specificity scores from the CNN implementation are calculated and compared with the respective ones from traditional Machine Learning classifiers. Subsequently, the best-performing model is compared to the state-of-the-art methods. The performance of the models is evaluated in two schemes, namely, a 10-Fold Cross-Validation (CV) (10-CV) and a Leave-One-Subject-Out (LOSO) Cross-Validation (LOSO-CV).

### 2.1 Description of the Database

The CuPiD IMU dataset (Bachlin et al., 2009) contains 3-axis accelerometer and gyroscope data from inertial sensors attached on patient wrists (Mazilu et al., 2013), sampled at 128 Hz. Measurements from 18 patients are included with begin and end timestamps of FoGs, Stops, and Walking-with-Turns.

Subjects in the CuPiD IMU dataset have an age range from 49 to 89 years old (mean: 68.9 years, standard deviation: 10.2 years) and have been diagnosed with PD from 2 to 18 years (mean: 8.8 years, standard deviation: 4.6 years). Each patient performed a walking session with 180° and 360° turns, in wide or narrow trails with obstacles that stimulate FoG events in a controlled environment. During the session, subjects were being asked to perform tasks and walk through crowded hospital rooms. In total, 184 FoG episodes are labeled from clinicians in 11 out of 18 patients, referred as S1–S11. The data of the seven subjects that did not display any episodes during the protocol are not included in the analysis. Time periods in which patients stood and discussed with clinicians or voluntarily stopped are also included in the dataset and are labeled as “Stop.” Labeled FoG events in the dataset can occur from 0.11 s up to 98.8 s (mean: 9.12 s, standard deviation: 15.35 s). Most episodes are short, with 50.8% of them lasting less than 3 s and 64.7% less than 5 s. FoG duration range is an important factor for detecting the events, as it plays an important role in defining the interval of the window and the slide-step.

**FIGURE 1:**
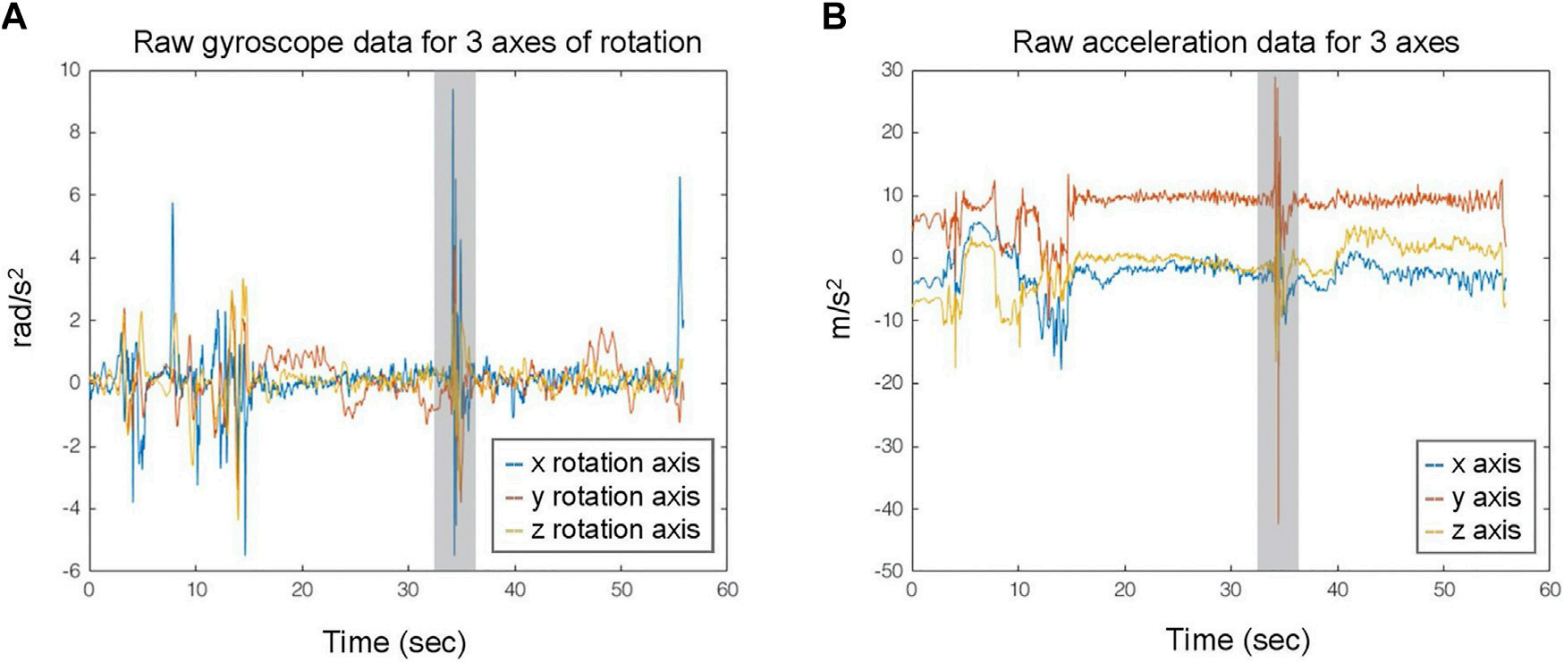
Exemplary data streams from triaxial gyroscope **(A)** and accelerometer **(B)** IMU recordings. The gray area indicates the FoG events.

An excerpt from the accelerometer and gyroscope data acquired from the S2 is depicted in Figure 1. In the latter, the occurrence of a FoG event (denoted with gray area) is evident near the 35s, exhibiting a distinguishable pattern during the walking activity, simultaneously appearing in both types of IMU data at 35s.

### 2.2 Preprocessing

Data three-dimensional (3D) IMU measurements are extracted for every PD patient from the CuPiD dataset. In particular, for each PD patient, the corresponding *N*-sample accelerometer and gyroscope data (sampled at $f_{s}=128$ Hz), i.e., ({**a**_*x*_: $\left[a_{x}[1\right]$, $a_{x}\left[2\right]$,…, $a_{x}\left[N\right]$]^*T*^}, {**a**_*y*_: $\left[a_{y}[1\right]$, $a_{y}\left[2\right]$,…, $a_{y}\left[N\right]$]^*T*^}, {**a**_*z*_: $\left[a_{z}[1\right]$, $a_{z}\left[2\right]$ …, $a_{z}\left[N\right]$]^*T*^}) and ({**g**_*x*_: $\left[g_{x}[1\right]$, $g_{x}\left[2\right]$,…, $g_{x}\left[N\right]$]^*T*^}, {**g**_*y*_: $\left[g_{y}[1\right]$, $g_{y}\left[2\right]$,…, $g_{y}\left[N\right]$]^*T*^}, {**g**_*z*_: $\left[g_{z}[1\right]$, $g_{z}\left[2\right]$ …, $g_{z}\left[N\right]$]^*T*^}) respectively, were constructed. The data are processed and formatted as a N×6 matrix for every PD patient, i.e., $S_{j}$ = [**a**_*x*_,**a**_*y*_,**a**_*z*_,**g**_*x*_,**g**_*y*_,**g**_*z*_], j=1,…,11 (PD patients).

Further, a three-second ($W_{l}$=3 s) sliding window with 0.25 s overlap is being selected for processing the $S_{j}$ data. The duration of the window and the overlap have been chosen as a trade-off between the duration of FoG events and the time resolution needed to detect a differentiation in wrist movement (Mazilu et al., 2015a). Every element of $S_{j}$ is being normalized with the maximum value per vector across all PD patients and rounded to 4 decimal digits for smaller bit storage.

### 2.3 Segmentation and Training Set Construction

**FIGURE 2:**
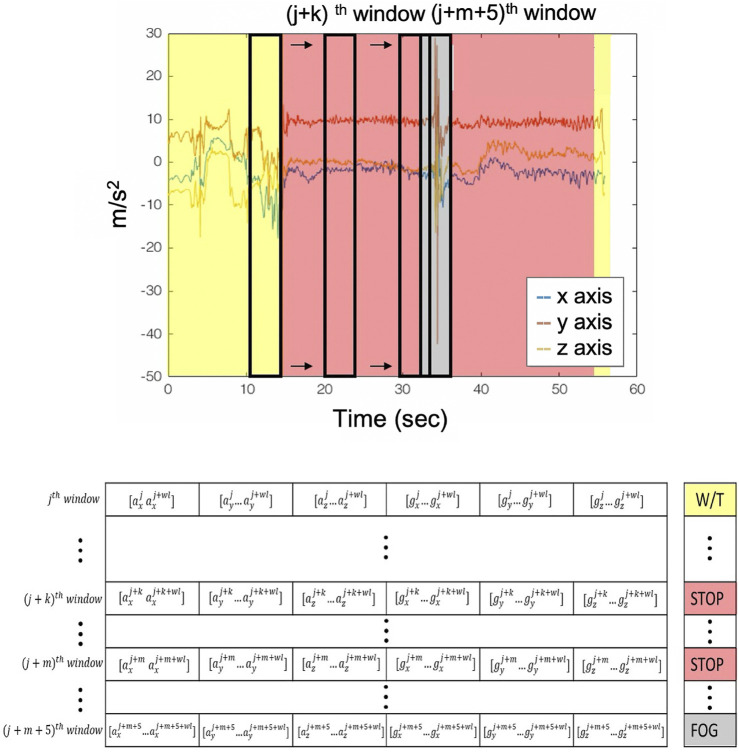
Sliding window and labeling procedure. Yellow areas are the walking with turn recordings; the red areas depict the stop event; and the gray ones depict the FoG event.

The resulted $S_{j}$ matrix is being reshaped to $K\times W_{l}\times 6$, where $K=\frac{N-W_{l}}{S_{l}}+1$ is the number of segments created. These segments were used to construct the training set for the CNN, labeled as “FoG” whether the FoG episode exists inside a window and whether a FoG event starts and ends inside this window. This was adopted so as to capture even short FoG events ($\leq W_{l}/2$). In the case where a FoG event is not completely integrated inside a window, the segment is being labeled as “FoG” only when the duration of the FoG is $\geq W_{l}/2$. Figure 2 illustrates the training set construction process.

### 2.4 Hyperparameter Tuning and Training of the CNN

**FIGURE 3:**
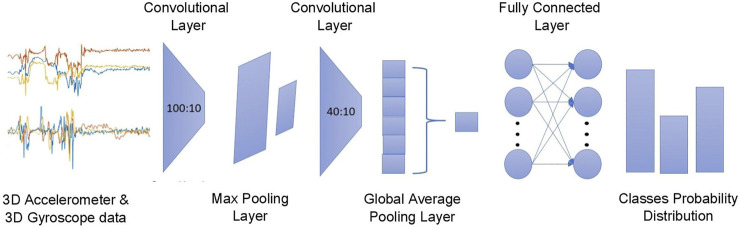
The proposed CNN architecture where the triaxial accelerometer and gyroscope streams are processed via a two-layer 1D convolution layer with six channels outputting the predictions regarding the probable outcomes.

Different CNN architectures are assessed by searching the best set of hyperparameters. The hyperparameters that were optimized include the depth of the network{2,3,4,5}, different kernel sizes [3–12], and number of filters [10–100]. The resulting CNN architecture, as depicted in Figure 3, consists of a two-layer one-dimensional (1D) convolution that processes the matrix $S_{j}$ with 100 and 40 filters, respectively, and a kernel size of 10, followed by a fully connected layer with an output of three nodes (i.e., “FoG,” “Stop,” “Walking-with-turns”). The first convolutional layer is being followed by a max-pooling layer, keeping the maximum value out for every three weights, in order to reduce the complexity and avoid over-fitting. For the second pooling layer, instead of the maximum, the average-pooling is used and the value of two weights is being taken. In this respect, only one weight remains per feature detector and the over-fitting is further prevented (Cook, 2017). Before the dense layer, a 50% dropout is applied to make the model intolerant to small abnormalities of the data (Srivastava et al., 2014). The CNN network is optimized to minimize the categorical cross entropy loss using the ADAM optimizer (Kingma and Ba, 2014). To mitigate overfitting, the model was trained for optimal epochs values. For selecting the optimal value for epochs, the train and validation losses were calculated for each of the CNN models. Loss values were monitored by Early stopping call back function. When there was an increment observed in loss values, training came to halt and the respective value of epoch indicated the optimal selection.

### 2.5 Evaluation Scheme

To evaluate the performance of the proposed model, two approaches are investigated. Considering that the model detects a FoG episode whenever a single window is being classified, sensitivity and specificity of the FoG class are calculated in a 10-CV and LOSO CV manner, so as to test the ability of the network to detect FoG events on unseen data. In the case of 10-CV, the dataset is shuffled and then split into 10 groups. One group acts as the test dataset, while the rest is being used for training. This procedure is repeated 10 times for each group and the average metrics are calculated to describe the results of the evaluation method. The adopted LOSO CV scheme follows the same procedure as the 10-CV with the difference being that the dataset is not randomly split, but every group holds the data of a single patient, with the groups being 11 each for S1–S11, accordingly.

## 3 Results

**FIGURE 4:**
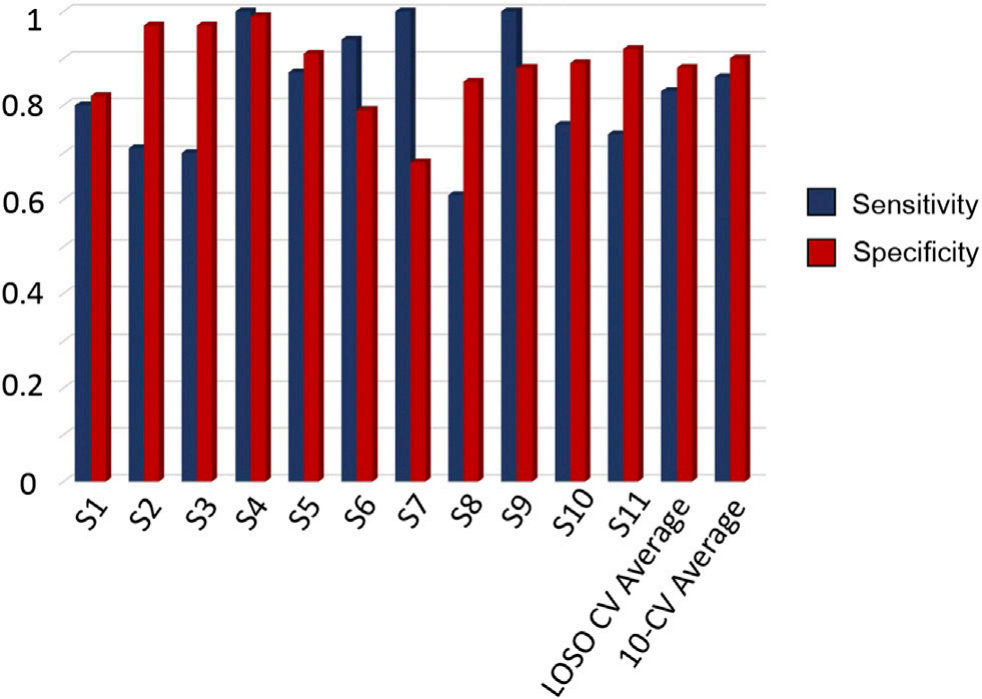
Resulted sensitivity and specificity scores per patient from LOSO CV, along with the average scores derived by the LOSO CV and 10-CV, based on single-window detection.

Figure 4 aggregates the metrics for the performance assessment of the two evaluation schemes, i.e., 10-CV and LOSO-CV. The comparisons between the CNN and the traditional classifiers are also presented below in Table 2. The specificity and sensitivity were reported to be equal to 88 and 83%, for LOSO-CV, and 90 and 86%, for 10-CV, respectively. Results for patient-specific sensitivity/specificity are presented in Figure 4. From the latter, it is observed that some patients’ FoGs present different trends in accelerometer and gyroscope data; hence, CNN performs poorly in recognizing them (61% sensitivity, S8 in Figure 4).

**TABLE 2:** Single-wrist comparison of sensitivity/specificity pairs for different analysis methodology. F: feature vector consisting of features of Mazilu et al. (2015b).

	10-CV		LOSO CV	
	Specificity	Sensitivity	Specificity	Sensitivity
F+Decision Tree	76%	71%	79%	63%
F+XGBoost	79%	81%	83%	70%
CNN (DeepFoG)	90%	86%	88%	83%

### 3.1 Performance of the Traditional Classifiers

To validate the assumption that a Deep Learning approach could result in better performance than traditional Machine Learning classifiers in detecting whether a window is FoG or not, this study compares the proposed CNN’s performance in training/testing with the performance of the Decision Trees and Extreme Boosting (XGboost) classification algorithms, incorporating the feature set proposed by Mazilu et al. (2015b) but coming from the single-wrist data. A higher performance is noticed from the CNN architecture (*see* sensitivity/specificity pairs in Table 2), hence, it is more suitable to be utilized for single-wrist detection.

### 3.2 Comparison of CNN With State-of-The-Art

Comparison between the DeepFoG performance and previously published approaches (with and without sensors mounted to the wrists of the patients) is presented in Table 1. Direct comparison is more relevant to the methods using sensors in the upper limb (Cole et al., 2011; Tripoliti et al., 2013; Mazilu et al., 2015b). It was demonstrated that DeepFoG has comparable classification performance with the related works in the literature which, however, use multiple sensors. The work by Camps et al. is of importance. The authors were the first to introduce a Deep Learning method for detecting FoG episodes in PD patients (Camps et al., 2018). In their approach, they used data collected by a waist-placed IMU (21 PD patients). Their proposed methodology achieved sensitivity/specificity equal to 92.6/88.7%. This study set a promising example for the use of Deep Learning in the accurate sensor-based detection of FoG episodes. Nonetheless, the use of a waist-placed sensor is likely to impose limitations related to the motion artifacts and inconvenience to the user. It is to be stressed that the comparison of our proposed method with the prior art cannot be direct and absolute, due to the different nature of the required inputs. Concretely, prior works rely on either multiple joint sensors or a single waist-placed sensor.

## 4 Discussion

Digital Health is an emerging field that could assist PD management via the realization of accessible tools towards detection and monitoring of the disease. Moreover, FoG episodes are strongly associated with the increased risk of fall and frailty of PD patients. Ultimately, the output of the trained DeepFoG, proposed in the current study, can then inform a timely application of RAS, combined with a hand vibration stimulus, to facilitate the PD patient to overcome the FoG event. The use of a smartwatch and the distinction between FoG events and the different natural movements of the user enables the transferability of the solution to a real-life environment. The latter reinforces the added value of the results towards capturing PD FoG episodes and provides objective information to the doctor regarding the frequency of the FoG event occurrence, towards objective monitoring of the PD patient. Moreover, the unobtrusive character of DeepFoG data acquisition contributes to the achievement of long-term adherence to the proposed solution, and enables the regulation of the medication towards reducing PD related risks.

DeepFoG may not outperform the related State-of-the-Art methods in both sensitivity and specificity metrics (Table 1). However, high performance is achieved by using only a single device (namely, smartwatch), when the others need at least two to arrive at a similar sensitivity/specificity. FoG events can be considered as rare events in the sensor streams during walking, so DeepFoG’s high specificity enables the ecological validity of the solutions. Moreover, the latency of DeepFoG to infer the FoG event is near real-time (considering 0.25 s time-window for each decision outcome); hence, even this retrospective study allows envisioning the timely automatic-RAS/hand vibration interventions. The DeepFoG can be integrated in a fused scheme with more PD risk-related variables, like heart rate variability features, age, or history of falls and FoGs, via a fuzzy-logic based model (Iakovakis et al., 2016), that could formulate an assisting feedback mechanism tailored to the everyday living activities of high-risk PD patients.

From a Deep Learning perspective, the use of the LOSO evaluation scheme informs us regarding the generalizability of the CNNs to detect FoG events for new subjects. It can also be observed that the 10-CV results were improved by 3 and 2% in specificity and sensitivity, when compared to the LOSO evaluation. Moreover, from the patient specific sensitivity/specificity pairs depicted in Figure 4, it can be observed that personalized cuing and FoG episodes could be detected via the use of personalized training for tuning the networks parameters to capture the patient-specific characteristics. The proposed version of DeepFoG was designed to classify three classes, “Walking with turns,” “Stop,”, and “FoG” achieving it with high *F*-score 0.81; instead of distinguishing just “FoG” and “Non FoG.” This makes DeepFoG insusceptible to false alarms FoG events due to intentional stopping, enhancing in this way its user friendliness and acceptability.

Despite the relatively high specificity and sensitivity estimated metrics, the dataset used to train and evaluate the CNN is quite limited (11 patients with FoG) and the FoG episodes are few (184). Hence, generalization of the outcomes should be performed with caution. Nevertheless, the recordings were longitudinal during the day and the detection results can be considered relevant and significant to encourage the continuation of the research towards evaluation of FoG detection-intervention using data from wearable devices. Importantly, we recognize that the optimal way to test a model’s generalization ability is by externally testing the model using an entirely new dataset. Given that such data are not currently available, we chose to perform a preliminary validation of the proposed framework before proceeding with a larger cohort. Provided that the preliminary evaluation gives promising results, we envision to continue with a larger population in our future research. Furthermore, lower prediction outcomes in some patients may raise questions about the various FoG patterns that exist in the examined cases and, thus, different gait patterns and their change along the PD severity need further investigation. Pilot tests on PD patients should be initialized in order to evaluate the quality and efficiency of the proposed system in home-based daily circumstances. Research effort towards this direction is already in progress.

## 5 Conclusion

The current study introduces a single-wrist-based FoG detection scheme that incorporates IMU data from a commercial smartwatch and CNN to facilitate a home-based falls prevention and FoG management. Instantaneous risk estimation enables the reduction of PD-related risks and can sustain the patient’s QoL. The Deep Learning-based method of single-arm might achieve similar accuracy as previously published methods, but with utilization of fewer sensors. The main advantage offered by our proposed methodology is its simplification and convenience attributed to the use of a single smartwatch rather than its improved accuracy. Nevertheless, our approach could be more preferable and easier accepted by the patients compared to the State-of-the-Art for feature extraction and classification methodology. Further evaluation of the proposed methodology on a large PD cohort in high risk remains to be conducted. Upon successful clinical validation, this work may provide an objective method to inform the doctors regarding the frequency of FoG events and it could test the effectiveness of the timely RAS/hand vibration interventions.

## Data Availability

The datasets generated for this study are available on request to the corresponding author.
